# Abnormal Vital Signs Predict Critical Deterioration in Hospitalized Pediatric Hematology-Oncology and Post-hematopoietic Cell Transplant Patients

**DOI:** 10.3389/fonc.2020.00354

**Published:** 2020-03-24

**Authors:** Asya Agulnik, Jeffrey Gossett, Angela K. Carrillo, Guolian Kang, R. Ray Morrison

**Affiliations:** ^1^Division of Critical Care, St. Jude Children's Research Hospital, Memphis, TN, United States; ^2^Department of Global Pediatric Medicine, St. Jude Children's Research Hospital, Memphis, TN, United States; ^3^Department of Biostatistics, St. Jude Children's Research Hospital, Memphis, TN, United States

**Keywords:** pediatric oncology, pediatric intensive care, Pediatric Early Warning System (PEWS), critical deterioration, emergency response systems, cardiopulmonary arrest

## Abstract

**Introduction:** Hospitalized pediatric hematology-oncology and post-hematopoietic cell transplant (HCT) patients have frequent deterioration requiring Pediatric Intensive Care Unit (PICU) care. Critical deterioration (CD), defined as unplanned PICU transfer requiring life-sustaining interventions within 12 h, is a pragmatic metric to evaluate emergency response systems (ERS) in pediatrics, however, it has not been investigated in these patients. The goal of this study was to evaluate if CD is an appropriate metric to assess effectiveness of ERS in pediatric hematology-oncology and post-HCT patients and if it is preceded by an actionable period of vital sign changes.

**Methods:** A retrospective review of all unplanned PICU transfers and floor cardiopulmonary arrests in a dedicated pediatric hematology-oncology hospital between August 2014 and July 2016. Vital signs and physical exam findings 48 h prior to events were converted to Pediatric Early Warning System-Like Scores (PEWS-LS) using cardiovascular, respiratory, and neurologic criteria.

**Results:** There were 220 deterioration events, with 107 (48.6%) meeting criteria for CD, representing a rate of 2.98 per 1,000-inpatient-days. Using the first event per hospitalization (*n* = 184), patients with CD had higher mortality (17.4 vs. 7.6%, *p* = 0.045), fewer median ICU-free-days (21 vs. 24, *p* = 0.011), ventilator-free-days (25 vs. 28, *p* < 0.001), and vasoactive-free-days (27 vs. 28, *p* < 0.001). Using vital sign data 48 h prior to deterioration events, those with CD had higher PEWS-LS on PICU admission (*p* < 0.001), spent more time with elevated PEWS-LS prior to PICU transfer (*p* = 0.008 to 0.023) and had a longer time from first abnormal PEWS-LS (*p* = 0.007 to 0.043). Significant difference between the two groups was observed as early as 4 h prior to the event (*p* = 0.047).

**Conclusion:** Hospitalized pediatric hematology-oncology and post-HCT patients have frequent deterioration resulting in a high mortality. In these patients, CD is over 13 times more common than floor cardiopulmonary arrests and associated with higher mortality and fewer event-free days, making it a useful metric in these patients. CD is preceded by a long duration of abnormal vital signs, making it potentially preventable through earlier recognition.

## Introduction

Delays in Intensive Care Unit (ICU) transfer for critically ill hospitalized patients have been consistently linked with worse outcomes including increasing organ dysfunction, longer ICU length of stay (LOS), and higher hospital mortality (1–4). Since the launch of the Institute for Healthcare Improvement's 100,000 Lives Campaign in 2004 (5, 6), timely identification of deterioration in hospitalized patients and appropriate activation of Emergency Response Systems (ERSs) has been an important element of hospital programs to reduce preventable harm and hospital mortality. Evaluating effectiveness of ERSs has been challenging in pediatrics, in part because cardiopulmonary arrest and death outside the Pediatric Intensive Care Unit (PICU) is rare in this patient population (7, 8). Critical deterioration (CD), defined as unplanned floor to PICU transfers requiring life-sustaining interventions within 12 h, has been proposed as a pragmatic metric to assess hospitals' ERSs, occurring 8-times more often than cardiopulmonary arrests and associated with mortality in a multidisciplinary pediatric setting (9).

Hospitalized pediatric hematology-oncology and post-hematopoietic cell transplant (HCT) patients are a unique patient population with more frequent deterioration and lower survival than other pediatric patients (7, 8, 10, 11). As in adults, there is evidence that delays in PICU transfer for these patients results in higher mortality (12). While tools to support ERSs, such as Pediatric Early Warning Systems (PEWS) (13–15), have been validated in this patient population, metrics to evaluate their effectiveness have not been studied. It is also unclear if CD in these patients is preceded by an actionable period of vital sign changes amenable to intervention by ERSs.

The aim of this study is to evaluate if CD is an appropriate metric to assess the quality and effectiveness of hospital emergency response systems in pediatric hematology-oncology and post-HCT patients, and whether CD is preceded by abnormal vital sign allowing for intervention.

## Materials and Methods

### Setting

St. Jude Children's Research Hospital is a 69-bed dedicated pediatric hematology-oncology hospital with an 8-bed PICU and 4-bed PICU-run intermediate care unit (IMCU) dedicated to this patient population. At the time of this study, St. Jude did not utilize a PEWS. On the hematology-oncology and HCT floors, vital signs are routinely taken by bedside nurses at least every 4 h in hospitalized patients, and more frequently in patients receiving certain therapies or those with signs of deterioration.

### Study Design

We conducted a retrospective review of all deterioration events among hospitalized patients (defined as unplanned PICU/IMCU admissions or floor cardiopulmonary arrests) between August 2014 and July 2016. Clinical characteristics, interventions, vital signs, and nursing physical exam documentation in the 48 h prior to the event, and patient outcomes were extracted from the electronic medical record. The deterioration event start was defined as the time of PICU transfer, operating room start time (emergency procedures resulting in unplanned PICU transfer), or the time of intubation or CPR initiation on the floor (floor respiratory or cardiac arrest). PIM2 and PRISM3 were calculated based on data entered by the institution into the Virtual PICU Systems database (VPS, LLC; Los Angeles, CA). These were missing in 6 events due to missing VPS entries. Hospital patient days were obtained from institutional statistics reports.

Critical deterioration (CD) was defined as an event requiring life-sustaining interventions (non-invasive or invasive mechanical ventilation, vasoactive infusions, or CPR) prior to or within 12 h of PICU admission. Event-free days (ICU-, vasoactive-, and mechanical ventilation-free days) were defined as the number of days in the first 28 days after ICU admission or event start when the patient was alive and without the intervention. Mechanical ventilation included invasive and non-invasive (CPAP and BiPAP) ventilation. Mortality was defined as occurring during the PICU stay.

### Evaluating Vital Sign Trends Prior to Deterioration Event and Calculation of PEWS-Like-Scores (PEWS-LS)

PEWS-like-scores (PEWS-LS) were calculated using vital signs data in the 48 h prior to PICU admission (see Supplemental Figures 1, 2 for the PEWS scoring tool used in analysis). The PEWS tool and vital sign limits were derived from those previously published (13, 15, 16), and vital sign limits were based on age-adjusted ranges for hospitalized children (16). The PEWS-LS was constructed by summing Cardiovascular (CV), Neurologic, and Respiratory scores in 15 min intervals. The CV score combined capillary refill and heart rate. The neurological score was based on level of consciousness. The respiratory score was based on oxygen use, oxygen saturation, and respiratory rate. The ranges of documented vital signs extracted from the medical record are presented in Supplemental Table 1. Neurologic, CV, and Respiratory sub-scores each had a possible range of 0–3, with 3 representing the most abnormal score. The PEWS-LS was a sum of these 3 sub-scores and had a theoretical range of 0–9. The PEWS-LS did not include all components of PEWS (nursing concern and family concern, physical exam elements not routinely documented with vital signs, see Supplemental Figure 1 for full PEWS tool), as these were not routinely documented with the frequency of vital sign assessments and our primary goal was to systematically evaluate the abnormality of vital sign changes. Documented values were carried forward until a change was noted in the medical record or patient was admitted to the PICU. For patients with <48 h between hospital admission and PICU transfer, only the available period of vital signs was used for analysis. When analyzing time from 1st abnormal PEWS-LS in the 12 h prior to event, only events with at least 6 h of documented vital sign data were included in analysis (10 events were excluded).

### Statistical Analysis

To control for multiple sampling, the first event per hospitalization was used for analysis, and events were treated as independent observations. Continuous and categorical variables were analyzed using appropriate summary statistics. Wilcoxon-Mann-Whitney, Pearson's chi-square, or Fisher's exact test were used to compare samples between groups. Trends in PEWS-LS in the 12 h prior to PICU admission were analyzed using a generalized estimating equation (GEE) Poisson model implemented in the GLIMMIX procedure of SAS (empirical option) with time as a restricted cubic spline with 4 knots (−11.5, −7.75, −4.25, and −0.5 h), CD as binary variables, and the two-way interaction. *P*-values were considered significant if < 0.05, with all *p*-values being 2-sided. *Post-hoc* comparisons were also made at 1 h intervals using a Holm-simulated adjustments for multiplicity. Analysis was performed using SAS software version 9.4 for Windows (SAS Institute Inc., Cary, NC).

## Results

During the study period, there were 220 deterioration events (2 floor cardiopulmonary arrests resulting in death prior to PICU transfer, 6 floor respiratory arrests requiring intubation and emergent PICU transfer, and 212 unplanned PICU transfers) among 160 unique patients and 184 unique hospital admissions, with a range of 1–4 hospital admissions per patient (see Supplemental Table 2). These occurred during 35,945 inpatient days (rate of 6.12 deterioration events per 1,000 inpatient days). Of these deterioration events, 107 (48.6%) met criteria for critical deterioration (CD), representing a rate of 2.98 CD events per 1,000 inpatient days, over 13 times more common than cardiopulmonary arrests on the floor (0.22 arrests per 1,000 inpatient days) in this setting. Twenty-nine events (13.2%) resulted in mortality (*n* = 2 floor cardiopulmonary arrests resulting in mortality prior to PICU admission and *n* = 27 unplanned PICU admissions resulting in mortality prior to PICU discharge). Characteristics of the 220 deterioration events are described in Supplemental Table 3.

Hospital admissions were taken as the primary unit of analysis. To control for multiple sampling, only the first deterioration event per hospitalization (*n* = 184) was used for analysis. Clinical characteristics and mortality of these deterioration events are described in Table 1. Patients had a median age of 10.6 years and events occurred a median of 3.3 days into their hospital course, resulting in a median PICU length-of-stay (LOS) of 3.4 days. Of these 184 deterioration events, 23 (12.5%) died prior to PICU discharge and 28 (15.2%) died prior to hospital discharge. Non-survivors were older, more likely to have a primary diagnosis of hematologic malignancy or be post allogenic hematopoietic cell transplant (HCT) (see Supplemental Table 4 for details regarding specific primary diagnoses). Non-survivors had higher risk of mortality on PICU admission as measured by PIM2 and PRISM3, had longer ICU LOS, and were more likely to require ICU-level interventions (high-flow nasal cannula (HFNC), non-invasive or invasive mechanical ventilation, vasoactive infusions, dialysis, and CPR) during their ICU course.

**Table 1:** PICU mortality in hospitalized pediatric hematology-oncology patients with deterioration.

**Characteristic**	**Total *n* = 184**	**ICU non-survivors *n* = 23**	**ICU survivors *n* = 161**	***p*-value**
Sex (M), *n* (%)	103 (56.0%)	16 (69.6%)	87 (54.0%)	0.161^a^
Age (years), median (IQR)	10.6 (3.0, 15.4)	13.5 (5.2, 18.7)	9.2 (2.9, 14.8)	0.050^b^
Primary Diagnosis, *n* (%)				0.010^c^
Hematologic malignancy	93 (50.5%)	19 (82.6%)	74 (46.0%)	
Solid tumor	71 (38.6%)	3 (13.0%)	68 (42.2%)	
Benign hematology	12 (6.5%)	1 (4.3%)	11 (6.8%)	
Other	8 (4.3%)	0	8 (5.0%)	
Post-HCT, *n* (%)				0.013^c^
Autologous	40 (21.7%)	11 (47.8%)	29 (18.0%)	
Allogenic	7 (3.8%)	0	7 (4.3%)	
No	137 (74.5%)	12 (52.2%)	125 (77.6%)	
Days from hospital admission to deterioration event, median (IQR)	3.3 (1.2, 11.3)	4.7 (1.8, 15.4)	3.2 (1.0, 10.6)	0.108^b^
PICU Admission Category, *n* (%)				0.121^c^
Respiratory	85 (46.2%)	16 (69.6%)	69 (42.9%)	
Cardiovascular	50 (27.3%)	2 (8.7%)	48 (29.8%)	
Neurologic	16 (8.7%)	2 (8.7%)	14 (8.7%)	
Fluid/electrolyte	5 (2.7%)	0	5 (3.1%)	
Other	28 (15.2%)	3 (13.0%)	25 (15.5%)	
Interventions during PICU course, *n* (%)				
HFNC	70 (38%)	14 (60.9%)	56 (34.8%)	0.016^a^
CPAP or BiPAP	33 (17.9%)	8 (34.8%)	25 (15.5%)	0.038^c^
Invasive mechanical ventilation	60 (32.6%)	17 (73.9%)	43 (26.7%)	<0.001^a^
Vasoactive infusions	64 (34.8%)	18 (78.3%)	46 (28.6%)	<0.001^a^
Dialysis	17 (9.2%)	10 (43.5%)	7 (4.3%)	<0.001^c^
CPR	9 (4.9%)	7 (30.4%)	2 (1.2%)	<0.001^c^
ICU LOS, median (IQR)	3.4 (1.5, 7.2)	15.5 (3.2, 32.5)	2.9 (1.5, 6.0)	0.004^b^
Hospital LOS, median (IQR)	17.3 (8.6, 38.0)	25.3 (7.1, 44.8)	17.1 (8.6, 37.4)	0.691^b^
	**Total** ***n*** **=** **179**	**ICU non-survivors** ***n*** **=** **20**	**ICU survivors** ***n*** **=** **159**	
PIM2, median (IQR)	3.1 (0.9, 5.2)	5.5 (4.1, 7.9)	1.8 (0.9, 4.8)	<0.001^b^
PRISM 3, median (IQR)	7.0 (3.0, 12.0)	11.5 (8.5, 14.5)	6.0 (3.0, 12.0)	0.002^b^

*BiPAP, Bilevel Positive Airway Pressure; CPAP, Continuous Positive Airway Pressure; CPR, Cardiopulmonary Resuscitation; HCT, Hematopoietic Cell Transplant; HFNC, High Flow Nasal Cannula; IQR, Inter-Quartile Range; LOS, Length of Stay; PICU, Pediatric Intensive Care Unit; PIM, Pediatric Index of Mortality; PRISM, Pediatric Risk of Mortality*;

a *Chi-squared*,

b *Wilcoxon*,

c *Fisher Exact*.

Of the 184 events used for analysis, 92 (50%) met criteria for CD (Table 2). Patients with CD were older and were more likely to be admitted to the PICU for cardiovascular issues (heart failure or shock). Rates of CD did not vary by primary diagnosis or with history of HCT. CD events had higher severity of illness (PIM2 and PRISM3) on PICU admission, required more PICU interventions during their ICU course with fewer ventilator- and vasoactive-free-days, had longer ICU LOS with fewer ICU-free-days, and had higher PICU mortality. These results were consistent across the HCT and non-HCT groups (please see Supplemental Table 5).

**Table 2:** Critical deterioration in hospitalized pediatric hematology-oncology patients.

**Characteristic**	**Critical deterioration *n* = 92**	**Not critical deterioration *n* = 92**	***p*-value**
Sex (M), *n* (%)	48 (52.2%)	55 (59.8%)	0.299^a^
Age (years), median (IQR)	12.5 (4.7, 16.1)	5.2 (2.1, 14.5)	0.002^b^
Primary diagnosis, *n* (%)			0.564^c^
Hematologic malignancy	51 (55.4%)	42 (45.7%)	
Solid tumor	32 (34.8%)	39 (42.4%)	
Benign hematology	6 (6.5%)	6 (6.5%)	
Other	3 (3.3%)	5 (5.4%)	
Post-HCT, *n* (%)			0.709^c^
Auto	22 (23.9%)	18 (19.6%)	
Allo	4 (4.3%)	3 (3.3%)	
No	66 (71.7%)	71 (77.2%)	
Days from hospital admission to deterioration event, median (IQR)	3.3 (1.1, 14.4)	3.1 (1.3, 9.5)	0.757^b^
ICU admission category, *n* (%)			<0.001^c^
Respiratory	41 (44.6%)	44 (47.8%)	
Cardiovascular	38 (41.3%)	12 (13.0%)	
Neurologic	7 (7.6%)	9 (9.8%)	
Fluid/electrolyte	1 (1.1%)	4 (4.3%)	
Other	5 (5.4%)	23 (25.0%)	
Interventions, *n* (%)			
HFNC	36 (39.1%)	34 (37.0%)	0.761^a^
CPAP or BiPAP	26 (28.3%)	7 (7.6%)	<0.001^a^
Invasive mechanical ventilation	46 (50.0%)	14 (15.2%)	<0.001^a^
Vasoactive infusions	57 (62.0%)	7 (7.6%)	<0.001^a^
Dialysis	12 (13.0%)	5 (5.4%)	0.075^a^
CPR	8 (8.7%)	1 (1.1%)	0.035^c^
ICU LOS, median (IQR)	4.1 (1.8,10.1)	2.6 (1.0,5.5)	0.015^b^
ICU-free days, median (IQR)	21 (8,25)	24 (18, 26)	0.011^b^
Hospital LOS, median (IQR)	20.7(10.2,40.9)	14.4(7.8,34.4)	0.119^b^
Vasoactive-free days, median (IQR)	27(25, 28)	28(28, 28)	<0.001^b^
Ventilator-free days, median (IQR)	25 (12.5, 28)	28(28,28)	<0.001^b^
Mortality, *n* (%)	16 (17.4%)	7 (7.6%)	0.045^a^
	**Critical Deterioration** ***n*** **=** **89**	**Not Critical Deterioration** ***n*** **=** **90**	
PIM2, median (IQR)	4.1(1.2,6.7)	1.1(0.9,4.2)	<0.001^b^
PRISM 3, median (IQR)	9.0(5.0,14.0)	5.0(0.0,10.0)	<0.001^b^

*BiPAP, Bilevel Positive Airway Pressure; CPAP, Continuous Positive Airway Pressure; CPR, Cardiopulmonary Resuscitation; HCT, Hematopoietic Cell Transplant; HFNC, High Flow Nasal Cannula; IQR, Inter-Quartile Range; LOS, Length of Stay; PICU, Pediatric Intensive Care Unit; PIM, Pediatric Index of Mortality; PRISM, Pediatric Risk of Mortality*.

a *Chi-squared*,

b *Wilcoxon*,

c *Fisher Exact*.

**Figure 1:**
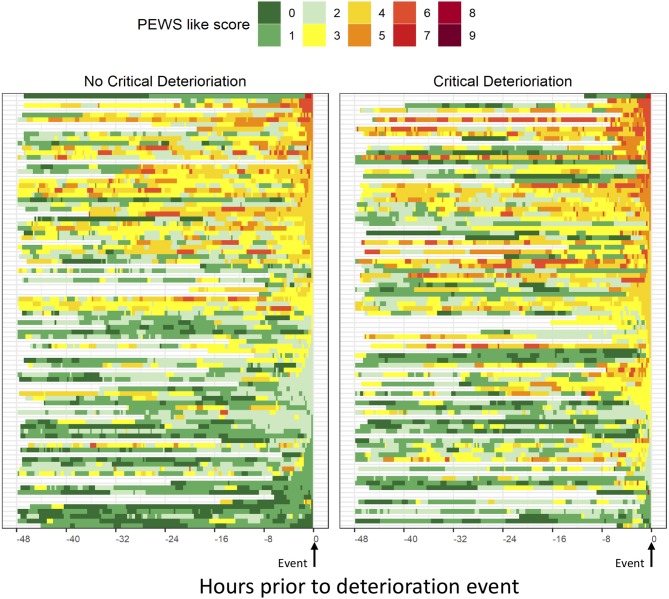
PEWS-Like-Score (PEWS-LS) in the 48 h prior to deterioration events with and without critical deterioration. PEWS-like-scores (PEWS-LS) were calculated using documented vital signs data in the 48-h prior to PICU admission or floor intervention using the PEWS tool and vital sign limits derived from those previously published (13, 15, 16). The PEWS-LS was constructed by summing cardiovascular (CV), Neurologic, and Respiratory scores in 15-min intervals. The CV score combined capillary refill and heart rate parameters. The neurological score was based on level of consciousness. The respiratory score was based on oxygen use, use, type, and flow of ventilation, oxygen saturation, and respiratory rate. Neurologic, CV, and Respiratory sub-scores each had a possible range of 0–3, with 3 representing the most abnormal score. The PEWS-like score was a sum of these 3 sub-scores and had a theoretical range of 0–9. Documented values were carried forward until a change was noted in the medical record or patient was admitted to the PICU. For patients with <48 h between hospital admission and PICU transfer, only the available period of vital signs was used for analysis.

**Figure 2:**
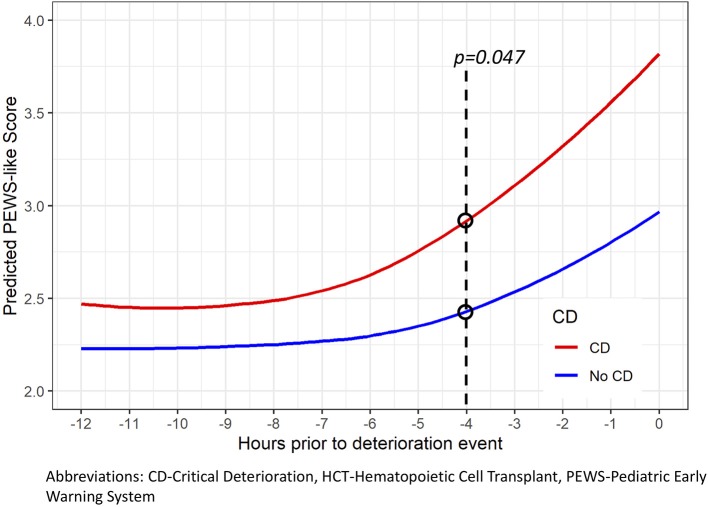
The predicted PEWS-Like-Score (PEWS-LS) over time prior to deterioration event in hospitalized pediatric hematology-oncology patients. Trends in PEWS-LS prior to PICU admission were analyzed using a generalized estimating equation (GEE) Poisson model implemented in the GLIMMIX procedure of SAS (empirical option) with time as a restricted cubic spline with 4 knots (−11.5, −7.75, −4.25, and −0.5 h), CD as binary variables, and the two-way interaction of time and CD as predictors. Both time (*p* < 0.001) and CD (*p* < 0.001) were significant predictors of PEWS-LS, but the two-way interaction was not significant (*p* = 0.26). At time of PICU transfer or intervention, the predicted mean PEWS-LS was 2.95 (95% CI 2.63, 3.31) for events without CD, and 3.81 (3.53, 4.11) for events with CD. Thus, the predicted PEWS-LS was 0.86 points higher in events with CD at the start of the event, *p* = 0.0003.

All 184 events had vital sign data available for analysis, with a similar duration of data in the 48 h prior to PICU transfer between those with and without CD (Table 3). Out of a maximum score of 9, the median PEWS-like-score (PEWS-LS) at time of PICU transfer among deterioration events was 4 (range 0–7), with higher scores among non-survivors (Supplemental Table 6) and those with CD (Table 3). The distribution of PEWS-LS in the 48 h prior to the deterioration event among those with and without CD is described in Figure 1. Using available vital sign data in the 48 h prior to the event, those with CD had PEWS-LS at the time of PICU transfer (*p* < 0.001), a higher maximum PEWS-LS in the 48 h prior to PICU transfer (*p* = 0.001), and spent a higher proportion of time with abnormal PEWS-LS prior to PICU transfer regardless of the PEWS threshold used for analysis (*p* = 0.008 to 0.023, Table 3). Using events with at least 6 h of preceding vital sign data, in the 12 h prior to PICU transfer or intervention, patients had an abnormal PEWS-LS (≥3) a median of 615 min (10.25 h) prior to their event, and this occurred 90 min earlier in those with CD (652.5 min with CD and 562.5 without, *p* = 0.043); this difference was more pronounced when higher PEWS-LS thresholds are used (217.5 min difference for PEWS-LS≥4, 397.5 min for PEWS-LS≥6, Table 3). Based on a Poisson GEE model fit using the 12 h prior to the event, both time (*p* < 0.0001) and CD (*p* < 0.0001) were significant predictors of PEWS-LS, but the two-way interaction was not significant (*p* = 0.26). For both events with and without CD, the predicted PEWS-LS increases as time approaches PICU transfer or intervention (Figure 2). Based on *post-hoc* tests at 1 h intervals, predicted PEWS-LS was higher in events with CD compared to those without CD starting at 4 h prior to PICU transfer or intervention (Holm-simulated adjusted *p* = 0.047).

**Table 3:** PEWS-like-score (PEWS-LS) and critical deterioration in hospitalized pediatric hematology-oncology patients.

	**All events**	**Critical deterioration**	**Not critical deterioration**	***p*-value**
	***n* = 184**	***n* = 92**	***n* = 92**	
Hours of available data prior in 48 h prior to event, median (IQR)	46.5 (28.0, 47.6)	46.3 (25.6, 47.5)	46.8 (31.0, 47.8)	0.235^a^
PEWS-LS at time of PICU transfer, median (IQR)	4 (2, 4)	4 (3, 5)	3 (1, 4)	<0.001^a^
Max PEWS-LS in 48 h prior to PICU transfer, median (IQR)	5 (4, 6)	5.5 (4, 6)	4 (4, 6)	0.001^a^
Percent of available time intervals in 48 h prior to PICU transfer with PEWS-LS ≥, median (IQR)				
PEWS-LS ≥3	34.6% (9.8%, 72.4%)	47.4% (14.0%, 76.7%)	27.2% (7.9%, 65.0%)	0.023^a^
PEWS-LS ≥4	10.9% (1.8%, 35.1%)	15.2% (3.2%, 39.8%)	8.9% (0.5%, 31.2%)	0.045^a^
PEWS-LS ≥5	1.5% (0%, 11.1%)	3.0% (0%, 13.7%)	0% (0%, 9.5%)	0.008^a^
	**Events with ≥6 h vital sign data**	**Critical deterioration**	**Not critical deterioration**	***p*****-value**
	***n*** **=** **174**	***n*** **=** **84**	***n*** **=** **90**	
Time (minutes) in 12 h prior to PICU transfer from first abnormal PEWS-LS, median (IQR)^*^				
PEWS-LS ≥3	615.0 (225.0, 720.0)	652.5 (367.5, 720.0)	562.5 (150.0, 720.0)	0.043^a^
PEWS-LS ≥4	540.0 (0.0, 720.0)	622.5 (90.0, 720.0)	405.0 (0.0, 720.0)	0.028^a^
PEWS-LS ≥5	60.0 (0.0, 720.0)	397.5 (0.0, 720.0)	0.0 (0.0, 705.0)	0.007^a^

* *Only events with at least 6 h of vital sign data prior to event start were included in this analysis (10 events excluded due to lack of adequate vital sign data)*.

a *Wilcoxon*.

*IQR, Inter-Quartile Range; PEWS-LS, Pediatric Early Warning System-Like-Score*.

## Discussion

Hospitalized pediatric hematology-oncology and post hematopoietic cell transplant (HCT) patients have frequent deterioration requiring PICU transfer or floor intervention resulting in high mortality (a rate of 6.12 events per 1,000 inpatient days and a PICU mortality of 13.2% in this study). Despite this frequency of deterioration, cardiopulmonary arrests in these patients were still uncommon (0.22 per 1,000 inpatient days). Critical deterioration (CD), defined as an event requiring life-sustaining interventions (non-invasive or invasive mechanical ventilation, vasoactive infusions, or CPR) prior to or within 12 h of PICU admission, occurred more frequently, with a rate of 2.98 events per 1,000 inpatient days, representing twice the rate in this patient population compared with that reported in a large multidisciplinary pediatric hospital (1.52 events per 1,000 inpatient days) (9).

In this study, CD was over 13 times more common than cardiopulmonary arrests on the floor, and associated with increased mortality, higher ICU LOS, more use of ICU interventions, fewer event-free days, and higher risk of mortality (PIM2, PRISM3). Because CD is more common than floor arrests and associated with poor outcomes and high PICU utilization, these findings support the use of CD to evaluate emergency response systems (ERSs) and hospital quality of care in pediatric hematology-oncology and post-HCT patients. While history of HCT is a known risk factor for mortality in critical illness and correlated with PICU mortality in our study, these patients did not have a higher rate of CD. These findings suggest that decisions around timing of PICU transfer were likely similar for pediatric oncology and post-HCT patients in our hospital, and CD is a reasonable quality measure in both patient populations.

Beyond demonstrating the relationship between CD and poor outcomes, we sought to explore if CD events are preceded by an actionable period of abnormal vital signs, making them amenable to prevention by ERS activation. Our analysis of the 48 h prior to PICU transfer or intervention in these patients demonstrates that events with CD have significantly longer and more severe durations of vital sign changes than those without CD. Notably, patients with CD spent nearly twice the amount of time with abnormal vital signs than those without CD, regardless of the PEWS-LS threshold used in analysis. In the 12 h prior to the event, patients had documented abnormal vital signs over 10 h prior to the event, with this occurring 1.5 h earlier in those with CD; this “lead time” increased with the magnitude of vital sign abnormality (higher PEWS-LS). Similarly, the vital signs were significantly more abnormal in patients with CD, compared to those without, 4 h prior to PICU transfer or intervention. These findings suggest that CD in pediatric hematology-oncology and post-HCT patients is preceded by a prolonged period of vital sign abnormalities on the floor, which may not be appropriately recognized or acted upon by the floor team. While prior studies have demonstrated an actionable period of abnormal vital signs prior to acute deterioration events in hospitalized general pediatrics patients (17), no study has previously examined this concept in hospitalized pediatric hematology-oncology patients which differ in their pathophysiology, risk of deterioration, and mortality from other pediatric populations. Similarly, the original description of CD in a large multidisciplinary hospital mentioned pediatric oncology patients but did not describe rates or characteristics of CD in this patient population (9). The limited available data in pediatric oncology patients demonstrates an impact of PICU transfer delay on mortality (12). It is possible that the prolonged period of abnormal vital signs prior to PICU transfer identified in our study represents a delay in identification and intervention for deterioration, partially contributing to the poor outcomes observed in these patients.

Previous studies in a large multidisciplinary pediatric hospital demonstrated that implementation ERS triggered by PEWS can reduce the frequency of CD (18). Our data suggest that systems to improve early identification of deterioration and vital sign changes, including PEWS, may also improve outcomes in these high-risk patients, despite conflicting evidence on the impact of PEWS in general pediatric settings with lower baseline rates of deterioration and mortality (19). Track-and-trigger systems (TTS) based on frequent assessment for and identification of abnormal vital signs in hospitalized children, such as multi-component rule-based systems designed around a scoring tool including vital signs (like PEWS), or more complex machine-learning systems based on the electronic medical record documented vital signs and other clinical data (20–22), may aid in better identification of patients at risk for deterioration and support clinical decision making around their disposition, particularly in this patient population. Such multi-component systems have consistently been shown to out-perform single-parameter TTS (20, 23) at identifying patients with deterioration, and our study suggests these findings hold true in pediatric hematology-oncology patients. Importantly, such systems must be associated with a robust response algorithm to assure identified abnormal vital signs are appropriately assessed and managed by the medical team. Similarly, routine measurement of CD in hospitals who manage hospitalized pediatric-oncology patients, along with the typical monitoring of cardiopulmonary arrests occurring outside the PICU, may improve assessment of the effectiveness of rapid response systems for these patients. This finding is particularly timely, as the development of PEWS to aid in recognition of critical illness in pediatric cancer patients outside the PICU was recently identified as the second most important research priority in pediatric onco-critical care through a large international expert concensus (24). There currently exist several PEWS (scoring tools associated with an escalation algorithm) that have been validated in pediatric hematology-oncology patients across a range of clinical settings (13–15), and we hope these findings will encourage the expansion of their use to support early identification of intervention for clinical deterioration in this patient population, leading to improved outcomes.

This study has several limitations. This is a single-center study in a pediatric hematology-oncology hospital. In the 2 years of study, we observed only 220 deterioration events. With the observed mortality of 13.2% we were not adequately powered to show a relationship between abnormal vital signs and mortality in our analysis (hence the need for a proxy metric such as CD). Despite the relatively small sample size, this represents one of the largest studies of deterioration in hospitalized pediatric hematology-oncology and post-HCT patients in the published literature, and our center is uniquely positioned to conduct this type of analysis in this patient population.

This is a retrospective study and is subject to typical limitations of this study design. Characteristics of deterioration events and vital sign data were extracted from documentation in the clinical chart. Due to criteria for entry into VPS, six events had no PIM2 or PRISM3 for analysis and 10 events had <6 h of preceding vital sign data. This, however, represents a small minority of documented events. Similarly, PEWS-LS were calculated based on vital sign and physical exam data documented by the bedside nurse during routine assessment; due to close proximity from hospital admission to PICU transfer, not all patients had 48 h of data available for analysis. To account for intermittent vital sign documentation, we carried forward documented data until a new observation was recorded. It is possible that this method missed more frequent vital sign changes than that which was documented by the beside nurse in the electronic medical record. However, as these patients were actively deteriorating during this time period, vital signs were likely worsening, rather than improving, and this method likely underestimated the true duration of vital sign abnormalities, making these results a conservative estimate of the true extent of abnormalities prior to PICU transfer or floor intervention.

The PEWS-LS was calculated using cardiovascular, respiratory, and neurologic vital signs, and physical exam findings. Some PEWS scoring tools validated in pediatric hematology-oncology patients use the added criteria of “nursing concern” and “family concern” to augment the objective components of the scoring tool (13, 15). Adding these components to the calculated PEWS-LS was not possible due to the retrospective nature of this study. Despite this limitation, however, the PEWS-LS was able to differentiate between events with and without CD, and higher PEWS-LS on PICU admission were associated with higher mortality, suggesting this approach was valid despite this limitation.

Finally, we can only speculate that the long duration of abnormal vital signs prior to CD suggests these events are potentially preventable through earlier intervention. However, there is an abundance of evidence that delays in ICU transfer for patients with clinical changes increases mortality (1–4, 12), and it is reasonable to hypothesize that earlier identification, intervention, and PICU transfer would in turn improve outcomes. This conclusion would need testing through prospective studies evaluating the implementation of PEWS or other systems to guide ERS activation in this high-risk patient population.

## Conclusion

Hospitalized pediatric hematology-oncology and post-HCT patients have frequent deterioration resulting in a high mortality. Critical deterioration in these patients is over 13 times more common than floor cardiopulmonary arrests and is associated with higher mortality and fewer event-free days, making it a useful metric for evaluating the quality of inpatient care and performance of emergency response systems. A protracted period of unrecognized abnormal vital signs on the floor prior to PICU transfer or floor intervention in these patients may be contributing to high rates of CD and high mortality. Systems for early identification of vital sign changes may improve hospital outcomes in these high-risk patients.

## Data Availability Statement

The datasets generated for this study are available on request to the corresponding author.

## Ethics Statement

This study was approved by the Institutional Review Board at St. Jude Children's Research Hospital. Written informed consent was not required to participate in the study in accordance with national legislation and institutional requirements.

## Author Contributions

Conceptualization and funding acquisition: AA and RM. Data curation, project administration, and validation: AA, JG, AC, and GK. Formal analysis, visualization, and writing—original draft: AA, JG, and GK. Investigation and methodology: AA, JG, GK, and RM. Resources: AA, GK, and RM. Software: JG and GK. Supervision: GK and RM. Writing—review and editing: AA, JG, AC, GK, and RM. All authors have reviewed and approved the final manuscript as submitted and agree to be accountable for all aspects of the work.

### Conflict of Interest

The authors declare that the research was conducted in the absence of any commercial or financial relationships that could be construed as a potential conflict of interest.
