# Supplementary material for: Abnormal Vital Signs Predict Critical Deterioration in Hospitalized Pediatric Hematology-Oncology and Post-hematopoietic Cell Transplant Patients
Source: Front Oncol. 2020 Mar 24;10:354. doi: 10.3389/fonc.2020.00354 (PMC7105633; doi:10.3389/fonc.2020.00354)
Supplement: Supplementary file 1 [file Data_Sheet_1.PDF]

Abnormal Vital Signs Predict Critical Deterioration in Hospitalized Pediatric Hematology-Oncology and Post-Hematopoietic Cell Transplant Patients

Asya Agulnik, MD, MPH[1,2]; Jeffrey Gossett[3]; Angela Carrillo[2], PhD; Guolian Kang[3], PhD; R. Ray Morrison, MD[1]

Supplemental Material

Table of Context

Item	Page
Supplemental Figure 1: PEWS scoring tool used to create PEWS-Like-Score	2
Supplemental Figure 2: PEWS vital sign limits used to create PEWS-Like-Score	3
Supplemental Table 1: Documented vital signs used for PEWS-Like-Scores	4
Supplemental Table 2: Hospital admission per individual patient	4
Supplemental Table 3: Characteristics of deterioration events in hospitalized pediatric hematology-oncology patients	5
Supplemental Table 4: Primary oncologic diagnosis, mortality, and critical deterioration	6
Supplemental Table 5: Critical Deterioration in Hospitalized Pediatric Hematology-Oncology Patients (among non-HCT and HCT patients)	7
Supplemental Table 6: PEWS-Like-Scores at time of PICU transfer and PICU mortality	8

Supplemental Figure 1: PEWS scoring tool used to create PEWS-Like-Score (PEWS-LS)

	0	1	2	3	Score
**Behavior/Neuro**	○ Playing/sleeping<br>○ Alert at patient's baseline	○ Sleepy when not disturbed (oriented when awake but if left alone falls back to sleep)	○ Irritable, difficult to console or arouse<br>○ Increase in patient's baseline seizure activity	○ Lethargic, confused, floppy<br>○ Reduced response to pain<br>○ Prolonged or frequent seizures<br>○ Pupils asymmetric or sluggish	
**Cardiovascular**	○ Normal heart rate for age<br>○ Skin tone appropriate for patient<br>○ Capillary refill ≤ 2 seconds	○ Mild tachycardia*<br>○ Pale<br>○ Capillary refill 3 seconds	○ Moderate tachycardia*<br>○ Grey<br>○ Capillary refill 4-5 seconds	○ Severe tachycardia*<br>○ Symptomatic bradycardia<br>○ Mottled<br>○ New onset/increase in ectopy, irregular heart rhythm or heart block<br>○ Capillary refill> 5 seconds	
**Respiratory**	○ Normal respiratory rate for age<br>○ No retractions	○ Mild tachypnea*<br>○ Mild increased work of breathing (flaring, retracting)<br>○ <5L via OxyMask™<br>○ Up to 1L NC >patient's baseline need<br>○ Increase in Fi02 via trach collar < 35% - 40%<br>○ Mild desaturation* (< 5 below patient's baseline)<br>○ Intermittent apnea self-resolving	○ Moderate tachypnea*<br>○ Moderate increased WOB (flaring retractions, grunting, use of accessory muscles)<br>○ >5L or ≤10L via OxyMask™<br>○ 2L NC > patient's baseline need<br>○ FiO2 via trach collar 40 – 55%<br>○ Moderate desaturation* (< 10 below patient's baseline)<br>○ Apnea requiring repositioning or stimulation<br>○ Nebs q 1-2 hour	○ Severe tachypnea*<br>○ New onset RR below normal for age<br>○ Severe increased WOB (head bobbing, paradoxical breathing)<br>○ >10L via OxyMask™ or requires non-rebreather<br>○ > 2L NC> patient's baseline need<br>○ FiO2 via trach collar > 60%<br>○ Severe desaturation* (<15 below patient's baseline)<br>○ Apnea requiring interventions other than repositioning or stimulation<br>○ Nebs every 30 min – 1 hour	

Supplemental Figure 2: Heart Rate and Respiratory Rate Limits for calculating PEWS-Like-Scores (PEWS-LS)

Heart Rate for Children

	Increase in Heart Rate			
**Age**	**Normal per minute**	**Mild 1**	**Moderate 2**	**Severe 3**
< 3 mos.	119-164	165-171	172-186	≥ 187
3 mos.- 5 mos.	114-159	160-167	168-182	≥ 183
6 mos.- 8 mos.	110-156	157-163	164-178	≥ 179
9 mos.- 11 mos.	107-153	154-160	161-176	≥ 177
12 mos.- 17 mos.	103-149	150-157	158-173	≥ 174
18 mos.- 23 mos.	98-146	147-154	155-170	≥ 171
2 yrs.	93-142	143-150	151-167	≥ 168
3 yrs.	88-138	139-146	147-164	≥ 165
4 yrs.- 5 yrs.	83-134	135-142	143-161	≥ 162
6 yrs.- 7 yrs.	77-128	129-137	138-155	≥ 156
8 yrs.- 11 yrs.	72-120	121-129	130-147	≥ 148
12 yrs. - 14 yrs.	66-112	113-121	122-138	≥ 139
15 yrs. - 18yrs.	62-107	108-115	116-132	≥133
> 18 yrs.	51-100	101-110	111-129	≥130

Respiratory Rate for Children

	Increase in Respirations			
**Age**	**Normal per minute**	**Mild 1**	**Moderate 2**	**Severe 3**
< 3 mos.	30-56	57-62	63-76	≥ 77
3 mos.- 5 mos.	28-52	53-58	59-71	≥ 72
6 mos.- 8 mos.	26-49	50-54	55-67	≥ 68
9 mos.- 11 mos.	24-46	47-51	52-63	≥ 64
12 mos.- 17 mos.	23-43	44-48	49-60	≥ 61
18 mos.- 23 mos.	21-40	41-45	46-57	≥ 58
2 yrs.	20-37	38-42	43-54	≥ 55
3 yrs.	19-35	36-40	41-52	≥ 53
4 yrs.- 5 yrs.	18-33	34-37	38-50	≥ 51
6 yrs.- 7 yrs.	17-31	32-35	36-46	≥ 47
8 yrs.- 11 yrs.	16-28	29-31	32-41	≥ 42
12 yrs. - 14 yrs.	15-25	26-28	29-35	≥ 36
15 yrs. - 18yrs.	14-23	24-26	27-32	≥ 32
> 18 yrs.	12-20	21-24	25-29	≥ 30

Supplemental Table 1: Documented vital signs used for PEWS-Like-Scores

Variable	n	Mean	SD	Median	IQR
Heart Rate	6419	129.51	25.32	130	112 to 145
Respiratory Rate	5684	30.48	12.5	28	22 to 37
SpO2	6289	96.29	3.84	97	95 to 99

Abbreviations: IQR-Inter-Quartile Range; PEWS-Pediatric-Early-Warning System; SD-Standard Deviation; SpO2-Oxygen Saturation

Supplemental Table 2: Hospital admissions per individual patient

Number of Hospital Admissions per Patient	Frequency	Percent
**1**	141	88.1%
**2**	15	9.4%
**3**	3	1.9%
**4**	1	0.6%

Supplemental Table 3: Characteristics of deterioration events in hospitalized pediatric hematology-oncology patients

Characteristic		Total n=220
Sex (M), n (%)		125 (56.8%)
Age (years), median (IQR)		10.6(3.0,15.5)
Primary Diagnosis		
	Hematologic Malignancy	120 (54.5%)
	Solid Tumor	79 (35.9%)
	Benign Hematology	12 (5.5%)
	Other	9 (4.1%)
Post-HSCT, n (%)		
	Auto	62 (28.2%)
	Allo	10 (4.5%)
	No	148 (67.3%)
Days from hospital admission to deterioration event, median (IQR)		4.3(1.4,19.3)
ICU Admission Category		
	Respiratory	104 (47.3%)
	Cardiovascular	55 (25.0%)
	Neurologic	20 (9.1%)
	Fluid/Electrolyte	7 (3.2%)
	Other	34 (15.5%)
Interventions during PICU course		
	HFNC	86 (39.1%)
	CPAP or BiPAP	40 (18.2%)
	Invasive Mechanical Ventilation	75 (34.1%)
	Vasoactive Infusions	73 (33.2%)
	Dialysis	24 (10.9%)
	CPR	12 (5.5%)
ICU LOS, median (IQR)		3.6 (1.6,8.2)
ICU-free days, median (IQR)		23.0 (14.0,25.0)
Hospital LOS, median (IQR)		21.9 (9.8,58.7)
Vasoactive-free days, median (IQR)		28.0 (26.0,28.0)
Ventilator-free days, median (IQR)		28.0 (22.0,28.0)
Critical Deterioration (CD), n (%)		107 (48.6%)
ICU Mortality, n (%)		29 (13.2%)
**Characteristic**		**Total n=214**
PIM2, median (IQR)		3.6 (1.0,5.6)
PRISM 3, median (IQR)		9.0 (4.0,12.0)

Supplemental Table 4: Primary oncologic diagnosis, mortality, and critical deterioration

Primary Diagnosis	Diagnosis Category	Total n=184	Critical Deterioration* n= 92	PICU non-survivors# n=23
ALL	Hematologic Malignancy	47 (25.5%)	25 (27.2%)	11 (47.8%)
AML	Hematologic Malignancy	32 (17.4%)	18 (19.6%)	6 (26.1%)
Lymphoma	Hematologic Malignancy	11 (6.0%)	6 (6.5%)	2 (8.7%)
Other Heme Malignancy	Hematologic Malignancy	3 (1.6%)	2 (2.2%)	
CNS tumor	Solid Tumor	21 (11.4%)	11 (12.0%)	
Neuroblastoma	Solid Tumor	21 (11.4%)	7 (7.6%)	
Sarcoma	Solid Tumor	15 (8.2%)	8 (8.7%)	1 (4.3%)
Other Solid Tumor	Solid Tumor	14 (7.6%)	6 (6.5%)	2 (8.7%)
Benign Hematology	Benign Hematology	12 (6.5%)	6 (6.5%)	1 (4.3%)
Other	Other	8 (4.3%)	3 (3.3%)	

*p-value = 0.893 when comparing distribution of CD vs non-CD across diagnoses

#p-value = 0.076 when comparing distribution of survivors and non-survivors across diagnoses

Supplemental Table 5: Critical Deterioration in Hospitalized Pediatric Hematology-Oncology Patients (among non-HCT and HCT patients)

		Non-HCT			HCT		
**Characteristic**		**CD n=66**	**Not CD n=71**	**p-value**	**CD n=26**	**Not CD n=21**	**p-value**
Sex (M), n (%)		38 (57.6%)	41 (57.7%)	0.984[a]	10 (38.5%)	14 (66.7%)	0.054[a]
Age (years), median (IQR)		12.5 (4.0,15.6)	5.1 (2.0,13.3)	0.002[b]	12.4(5.2,16.8)	11.9(3.0,16.2)	0.500[b]
Days from hospital admission to deterioration event, median (IQR)		2.4 (0.7,4.7)	2.8 (1.3,5.6)	0.239[b]	18.6(9.0,30.5)	7.3(1.4,27.3)	0.219[b]
ICU Admission Category				<0.001[c]			0.089[c]
	Respiratory	25 (37.9%)	33 (46.5%)		16 (61.5%)	11 (52.4%)	
	Cardiovascular	31 (47.0%)	10 (14.1%)		7 (26.9%)	2 (9.5%)	
	Neurologic	6 (9.1%)	6 (8.5%)		1 (3.8%)	3 (14.3%)	
	Fluid/Electrolyte	0	4 (5.6%)		1 (3.8%)		
	Other	4 (6.1%)	18 (25.4%)		1 (3.8%)	5 (23.8%)	
Interventions							
	HFNC	20 (30.3%)	22 (31.0%)	0.931[a]	16 (61.5%)	12 (57.1%)	0.76[a]
	CPAP or BiPAP	15 (22.7%)	5 (7.0%)	0.009[a]	11 (42.3%)	2 (9.5%)	0.012[a]
	Invasive Mechanical Ventilation	28 (42.4%)	9 (12.7%)	<0.001[a]	18 (69.2%)	5 (23.8%)	0.002[a]
	Vasoactive Infusions	41 (62.1%)	4 (5.6%)	<0.001[a]	16 (61.5%)	3 (14.3%)	0.001[a]
	Dialysis	4 (6.1%)	4 (5.6%)	1.000[a]	8 (30.8%)	1 (4.8%)	0.030[c]
	CPR	5 (7.6%)	0	0.024[c]	3 (11.5%)	1 (4.8%)	0.617[c]
ICU LOS, median (IQR)		3.7 (1.3,6.8)	1.9 (0.9,4.1)	0.035[b]	6.4(2.9,17.9)	4.1(2.6,8.7)	0.280[b]
ICU-free days, median (IQR)		22 (12, 25)	25 (21, 26)	0.025[b]	14.0(0.0,23.0)	19.0(15.0,24.0)	0.369[b]
Hospital LOS, median (IQR)		16.1(7.4,29.0)	12.4(7.0,24.9)	0.381[b]	40.9(21.3,74.5)	35.4(14.6,77.9)	0.447[b]
Vasoactive-free days, median (IQR)		27 (25, 28)	28 (28, 28)	<0.001[b]	26.0(24.0,28.0)	28.0(28.0,28.0)	0.017[b]
Ventilator-free days, median (IQR)		27.5 (15, 28)	28 (28 ,28)	<0.001[b]	22.0(9.0,26.0)	28.0(25.0,28.0)	0.004[b]
Mortality, n (%)		9 (13.6%)	3 (4.2%)	0.052[a]	7 (26.9%)	4 (19.0%)	0.731[c]
		**CD n=63**	**Not CD n=69**	**p-value**	**CD n=26**	**Not CD n=21**	**p-value**
PIM2, median (IQR)		3.7 (1.1,6.0)	1.0 (0.9,3.6)	<0.001[b]	4.3(2.9,13.6)	4.0(1.1,4.8)	0.089[b]
PRISM 3, median (IQR)		8.0 (4.0,13.0)	5.0 (0.0,9.0)	0.003[b]	10.0(7.0,15.0)	7.0(3.0,12.0)	0.077[b]

Abbreviations: BiPAP-Bilevel Positive Airway Pressure, CD- Critical Deterioration, CPAP-Continuous Positive Airway Pressure, CPR-Cardiopulmonary Resuscitation, HCT-Hematopoietic Cell Transplant, HFNC-High Flow Nasal Cannula, IQR-Inter-Quartile Range, LOS-Length of Stay, PICU-Pediatric Intensive Care Unit, PIM-Pediatric Index of Mortality, PRISM-Pediatric Risk of Mortality;

[a]Chi-squared, [b]Wilcoxon, [c]Fisher Exact

Supplemental Table 6: PEWS-Like-Scores at time of PICU transfer and PICU mortality

Characteristic		Total n=184	ICU non-survivors n=23	ICU survivors n=161	p-value
PEWS-like score					0.033
	median (IQR)	4 (2, 4)	4 (3, 5)	3 (2, 4)	
	range	0~7	1~6	0~7	
CV score					0.681
	median (IQR)	1 (1, 2)	1 (1, 2)	1 (1, 2)	
	range	0~3	0~3	0~3	
Respiratory score					0.001
	median (IQR)	2 (1, 3)	3 (2, 3)	2 (1, 3)	
	range	0~3	1~3	0~3	
Neurologic score					0.277
	median (IQR)	0 (0, 0)	0 (0, 0)	0 (0, 0)	
	range	0~3	0~3	0~3	
